# Current status of herbal medicine research for respiratory diseases induced by particulate matter: a protocol for a scoping review

**DOI:** 10.1186/s13643-022-01904-9

**Published:** 2022-03-01

**Authors:** Jungtae Leem, Yohwan Kim, Kwan-Il Kim

**Affiliations:** 1 Research Center of Traditional Korean Medicine, Wonkwang University, 460, Iksan-daero, Sin-dong, Iksan, Jeollabuk-do 54538 Republic of Korea; 2 National Institute for Korean Medicine Development, 94, Hwarang-ro, Gyeongsan-si, Gyeongsangbuk-do 38540 Republic of Korea; 3 Division of Allergy, Immune and Respiratory System, Department of Internal Medicine, College of Korean Medicine, Kyunghee University, 26, Kyungheedae-ro, Dongdaemun-gu, Seoul, 02447 Republic of Korea

**Keywords:** Particulate matter, Herbal medicine, Medicine, traditional, Respiratory tract disease, *In vitro*, *In vivo*, Scoping review

## Abstract

**Background:**

Particulate matter (PM) is an important environmental risk factor for the initiation and exacerbation of respiratory disease. Various herbal medicines have exhibited a reduction in symptoms of respiratory diseases induced by PM in animal models. However, the types and characteristics of studies on herbal medicine for respiratory diseases by PM have not been reviewed. This scoping review will focus on the currents status and research gap of herbal medicines for respiratory diseases caused by PM.

**Methods:**

We will follow the scoping review framework developed by Arksey and O’Malley. MEDLINE (via PubMed), EMBASE, and the Cochrane Central Register of Controlled Trials will be searched for relevant English-language publications, and only peer-reviewed, controlled comparative *in vivo/in-vitro/human* studies examining the effects of herbs on respiratory disease induced by PM will be included. The basic characteristics, research methods, detailed regimens, possible mechanisms, outcomes, and results will be extracted using a predefined standardized data extraction form. Outcomes will be presented in the following categories: pulmonary function, inflammatory markers, reactive oxygen species, histology and mechanisms, and adverse events. Two researchers will independently perform the study selection, data extraction, and quality assessment. We will also present the research map and implications for further study.

**Ethics and dissemination:**

Ethical approval is not required because individual patient data will not be included. The findings will be disseminated through peer-reviewed publications or conference presentations.

**Systematic review registration:**

This review protocol has been registered with the Open Science Framework on February 12, 2021 (https://osf.io/s7uvk/)

**Supplementary Information:**

The online version contains supplementary material available at 10.1186/s13643-022-01904-9.

## Background

Particulate matter (PM) is a form of air pollution that can be inhaled, and comprises microscopic particles of varying size, composition, and origins. The fraction of particles suspended in the air that are of less than 10 μm in diameter is defined as ‘coarse PM’ (PM10), that of particles less than 2.5 μm in diameter as ‘fine PM’ (PM2.5), and that of particles less than 0.1 μm in diameter as ‘ultrafine PM’ (PM0.1) [1]. In recent years, global interest in PM has rapidly increased, and many studies on the health effects of PM exposure have been conducted, revealing adverse effects on the respiratory and cardiac systems [2–4]. PM is known to induce or exacerbate respiratory diseases because of its direct effects on the respiratory system. Larger-sized particles affect the upper respiratory tract, while particles less than 2.5 μm in diameter reach further down into the respiratory system affecting the lung interstitium and alveoli [5, 6]. Epidemiological reports have shown that PM10 can exacerbate asthma or chronic obstructive pulmonary disease (COPD) and induce inflammation of the respiratory system [7–10]. PM2.5 also has adverse health effects, and the increment in PM2.5 is associated with an increased mortality rate of the respiratory disease [11], and long-term exposure to PM2.5 can increase the COPD incidence [12]. Substantial medical costs are derived from air pollution and PM that increase the economic burden [13].

With increase in environmental pollution and the aging global population, the incidence and exacerbation of asthma and COPD owing to air pollution are bound to increase. Therefore, it is important to seek strategies for the prevention and alleviation of respiratory diseases caused by PM. However, there are currently no drugs specifically developed for the prevention or management of the damage caused by PM.

To overcome the limitations and lack of evidence of conventional medicines available for the prevention and/or management of respiratory symptoms caused by PM, complementary and alternative medicine (CAM) and herbal medicine treatment strategies have garnered increased interest. Several systematic reviews on the effects of herbal medicines for respiratory diseases, such as COPD, asthma, and lung cancer, have been published [14–16]. However, there are few studies on lung disease induced by PM. Although some animal model experimental studies conducted sporadically for respiratory diseases induced by PM have demonstrated the mechanisms and effectiveness of herbal medicine [17–20]; however, no conclusion was reached on the herbal medicine, mechanism, regimen, research design, and outcome appropriate for research on lung disease induced by PM. Therefore, there is an increasing need for a scoping review of the research design and characteristics of studies on herbal medicine for respiratory diseases by PM. An agreement on the research methods and protocols were not met between researchers. In general, much of the evidence available is traced back to studies on experimental animals since ethical concerns are associated with clinical trial designs in which patients are directly exposed to PM.

In this scoping review, we will summarize the experimental/clinical studies using herbal formulas or single herbs for respiratory diseases caused by PM. We will also explore the mechanisms of action of herbal treatments and the markers used in the studies. This summary of currently available research evidence will facilitate the design of further studies and provide detailed information of studies on herbal medicine for respiratory diseases by PM. These data will also be used as fundamental information for further clinical research and clinical practice.

## Methods

### Study design and registration

We will follow the scoping review methodology developed by Arksey and O’Malley [21] and other authors [22, 23]. This scoping review protocol complies with the Preferred Reporting Items for Systematic Reviews and Meta-Analyses (PRISMA) Extensions for Scoping reviews, (PRISMA-Scr) guidelines [23]. This review protocol has been registered with the Open Science Framework on February 12, 2021 (https://osf.io/s7uvk/).

### Purpose of choosing the scoping review methodology

We selected scoping review methodology according to previous guidance about selecting scoping and systematic reviews [24]. Moreover, we did not focus on the aims of a formal systematic review, including calculating the effect and harm of the intervention, quantitatively synthesizing data, assessing methodological quality, and deriving statements to guide decision-making. However, as it is an emerging topic, we planned to identify various available literature, describe adopted research methodology/intervention/outcomes, analyze mechanisms, and provide knowledge gaps, which are the general purposes of scoping review. This is the reason why we selected scoping review methodology.

### Stage 1: identifying the study questions

This stage was archived with a preliminary literature search on previous research and agreement of research team members for better scoping of research objectives. The research team comprised one respiratory disease specialist (KK), a specialist in clinical research on traditional East Asian medicine (JL), and a researcher on literature review (YK). Suggestions and revision of the research question were requested from other related experimental/clinical research experts. The following questions will be addressed in our scoping review following the consensus of the research team.

1. What is the popular herbal medicine used in the studies on lung disease induced by PM, and which herbal medicine is better for each indication?
2. What is the primary and secondary outcome reported in the studies on lung disease induced by PM?
3. What types of mechanisms were explored regarding the effect of herbal medicine for lung disease induced by PM?
4. What are the known adverse events of herbal medicine for lung disease induced by PM?
5. How long should herbal medicine be administered for animal/human studies? What is the pattern of efficacy change according to the administration period in animal/human studies?
6. What types of animal models are frequently used in experimental research and what are the strengths and limitations of each model?
7. What kinds of research designs were adopted in the clinical research?

### Stage 2: identifying relevant studies

#### Information source

We will restrict our scoping to peer-reviewed, English-language studies on herbal medicine for respiratory diseases induced by PM. The literature search will be conducted from the inception to the present (March 2021). The following databases will be searched: MEDLINE (via PubMed), EMBASE, and the Cochrane Central Register of Controlled Trials. We will also consider gray literature searches via Google Scholar, Open Gray, and ProQuest Dissertations and Theses Global. The reference lists of the retrieved articles and the relevant systematic reviews will be searched manually. Efforts will be made to get in touch with the authors of the published articles of which electronic files cannot be obtained. The search strategy was consulted with the librarian, an expert on scoping review, and a specialist on lung disease. We will use search terms related to PM and intervention. Related medical subject heading (MeSH) terms and synonyms in various combinations will be used in the search strategy. The terms to be used in relation to PM include “fine dust,” “PM,” and “coarse particle.” The terms to be used in relation to intervention include “herbal medicine” and “herb.” The search strategies are presented in supplementary digital content (Additional file 1: Appendix 1).

### Eligibility criteria: types of studies

Controlled, comparative, *in vivo/in vitro/human* studies examining the effects of herbs on respiratory diseases induced by PM will be included.

#### Eligibility criteria: types of interventions

Multiple or single medicinal herb preparations and fractions of medicinal herb preparations will be considered eligible as interventions. We will include herbal preparations of any type, such as liquids, gels, tablets, and extracts, but only those that are orally administered. Any type of comparative intervention will be included. The duration of the treatment period will not be restricted.

#### Eligibility criteria: types of outcome measurements

The last time-point acquired value will be extracted. The outcomes will be categorized as follows: (1) primary respiratory function assessed by tidal mid expiratory flow (EF_50_) [25], (2) other respiratory functions, such as tidal volume, respiratory rate, and respiratory minute rate time taken to inspire and expire, (3) inflammatory markers, (4) reactive oxygen species (ROS), and (5) histology and mechanisms. In terms of safety issues, we will also investigate adverse events and dropout rates.

### Stage 3: study selection

**Fig. 1:**
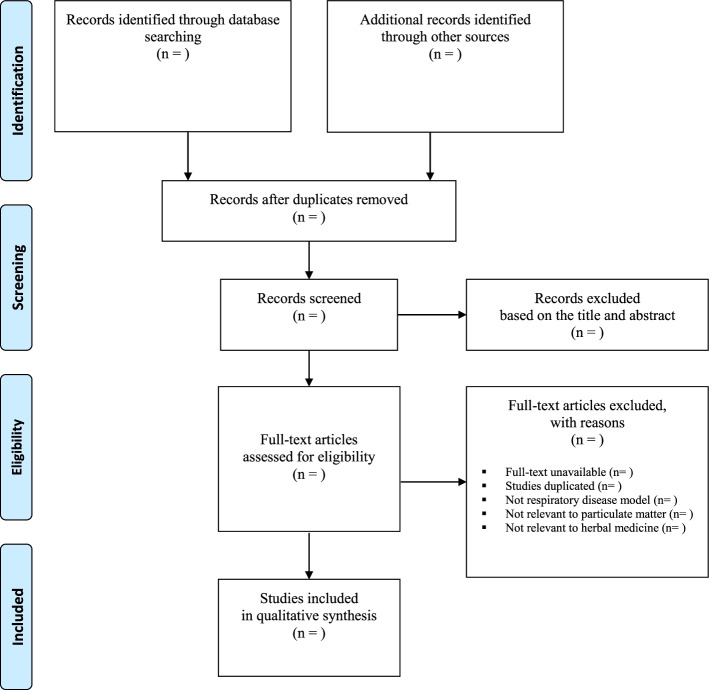
Flowchart of identification and screening for the eligible studies

Two reviewers (YK and JL) will independently conduct the entire study selection process. After performing database searches and eliminating duplicate publications, the titles and abstracts of the searched studies will be screened for inclusion. For the articles identified as potentially relevant, the full text will be checked to determine whether the study will be included. All articles will be included or excluded based on predetermined criteria, and the reviewers will record the reasons for exclusion. Any discrepancies will be resolved through discussion with another researcher. The details of the study selection procedure are presented in Fig. 1.

### Stage 4: charting the data

A preliminary data extraction sheet was developed with agreement on the pilot testing by the research team. After several revisions, a standardized data extraction form was developed.

The following items will be extracted from the included studies: general information, such as the last name of the first author and publication year; participants’ characteristics, such as sex and age, and disease duration and severity; for the animal studies, the following information will also be extracted: species, weight, and the respiratory models employed; methods for inducing respiratory disease; type of respiratory disease induced; details regarding the PM, such as type, origin, characteristics, and method by which it was obtained; herbal medicines characteristics, such as route of administration, composition, dosage, and treatment period; and details of the control intervention. Data regarding research results (effect and safety), research findings, and proposed mechanisms will be extracted.

The data extraction process will be performed independently by two reviewers (YK, JL); these reviewers will crosscheck data from all of the included studies. Any disagreement between the two reviewers will be resolved through discussion with another researcher (KIK).

### Stage 5: collating, summarizing, and reporting the results

Extracted data will be utilized to develop an analytical framework for collating, synthesizing, and summarizing the extracted data. In the qualitative analysis stage, we will provide a table named “Characteristics of included studies,” including the author, published year, country, age, sex, animal model, number of subjects, treatment group intervention (herbal medicine and composition) and dosage, control group intervention and dosage, and reported outcomes. We will also provide another table named “Effect, mechanism, and safety of herbal medicine for lung disease induced by PM.” The results of the experiment will be presented according to each intervention and dosage, possible mechanism suggested by the original research author, and the number of each adverse event. We will also gather research implications for further research from original articles that can be helpful for the research community. In addition, we will provide a figure named “Research map,” which will visualize possible mechanisms and candidate herbal preparation/medicinal plants. The research map can also help identify knowledge gaps of current studies on the topic. All the qualitative analysis processes will be conducted using Microsoft Excel.

## Discussion

This is the first scoping review on herbal medicine for lung diseases included by PM. Through this scoping review, we can identify available evidence and research methodology in this field. We can also analyze knowledge gaps for further studies. Particularly, we expect to present the effects, mechanisms, and preferred experimental protocol for herbal medicine. We adopted the scoping review methodology for several reasons. As we are focusing on the current research status on experimental and clinical research on herbal medicine for lung disease induced by PM, we can identify any type of available evidence in our field of interest by scoping review methodology [24]. We can also find knowledge gaps on the topic using a research map [24] and provide a comprehensive, detailed, and intuitive understanding of the topic’s research status via our scoping review.

The deterioration of respiratory function due to PM is a global concern. The onset or exacerbation of respiratory disease due to PM is a major challenge to human health. Therefore, in this scoping review of experimental and clinical studies, we aim to provide an overall scope of the available evidence, research methodology, and research gap of herbal medicines for treating respiratory diseases induced or worsened by PM. The search strategy has been established, and a conservative approach to data collection will be followed to avoid bias. For a broad review process, we will also consider gray literature. Moreover, predefined contributions and methods will enhance the robustness of our scoping review. If it is indicated, we will also consult experts in the field to increase the quality of the review.

This study has several limitations. We did not plan for the optional sixth step (consultation) in our review. Since it is difficult and unethical to artificially create a respiratory disease induced by PM in humans, there may not be many controlled clinical studies. Our study also has several strengths. We will provide the candidate mechanisms of herbal medicine that will enhance the quality of further research studies. It will also be helpful for a better understanding of the experimental/clinical data acquired from various research designs. This scoping review may guide researchers to plan their experiments/clinical study more effectively, thus reducing the resource burden for future research. It could also alleviate the advanced understanding of the mechanisms and target biomarkers of respiratory inflammation caused by PM exposure, ultimately assisting in overcoming the challenges involved in treating respiratory diseases induced by PM.

This research does not require ethical consideration and informed consent, as we will not use personal medical information but only information from previously published articles. For more dissemination, the research findings will be submitted to a scientific journal. This will also be presented at academic conferences. Moreover, we will also develop an e-leaflet to provide key findings of our review to disseminate via social network services to the research community.

## Supplementary Information

**Additional file 1: Appendix 1.** Search Strategy.

**Additional file 2: Appendix 2.** PRISMA-Scr Checklist.

## Data Availability

Data sharing is not applicable to this article as no datasets were generated or analyzed during the current study.

## Abbreviations

CAM: Complementary and alternative medicine
CENTRAL: Cochrane Central Register of Controlled Trials
CI: Confidence interval
COPD: Chronic obstructive pulmonary disease
MD: Mean difference
MeSH: Medical Subject Heading
PM: Particulate matter
PRISMA: Preferred Reporting Items for Systematic Reviews and Meta-Analyses
PRISMA-Scr: PRISMA Extensions for Scoping reviews
ROS: Reactive oxygen species
SYRCLE: Systematic Review Center for Laboratory Animal Experimentation
