# Supplementary material for: Current status of herbal medicine research for respiratory diseases induced by particulate matter: a protocol for a scoping review
Source: Syst Rev. 2022 Mar 1;11:35. doi: 10.1186/s13643-022-01904-9 (PMC8889652; doi:10.1186/s13643-022-01904-9)
Supplement: Supplementary file 1 — Additional file 1: Appendix 1. Search Strategy. [file 13643_2022_1904_MOESM1_ESM.docx]

**Appendix 1. Search strategies**

| **Medline (via Pubmed)** |
| --- |
| #1 "Ambient Particulate Matter" [TIAB] |
| #2 "Asian dust" [TIAB] |
| #3 "coarse particle" [TIAB] |
| #4 Dust [Mesh] |
| #5 "Fine dust" [TIAB] |
| #6 "Airborne Particulate Matter" [TIAB] |
| #7 "Fine particle" [TIAB] |
| #8 "particular matter" [TIAB] |
| #9 "Particulate Air Pollutants" [TIAB] |
| #10 "Particulate Matter" [Mesh] |
| #11 "Particulate Matter, Airborne" [TIAB] |
| #12 "Particulate Matter, Ambient" [TIAB] |
| #13 PM0.1 [TIAB] |
| #14 PM10 [TIAB] |
| #15 PM2.5 [TIAB] |
| #16 "Pollutants, Particulate Air" [TIAB] |
| #17 "Suspended particle" [TIAB] |
| #18 "ultrafine particle" [TIAB] |
| #19 ultrafine particulate [TIAB] |
| #20 Yellow dust [TIAB] |
| #21 suspended particulate matter [TIAB] |
| #22 "chinese drug" [TIAB] |
| #23 "chinese formulation" [TIAB] |
| #24 "chinese herb" [TIAB] |
| #25 "chinese medicine" [TIAB] |
| #26 "chinese plant" [TIAB] |
| #27 "chinese prescription" [TIAB] |
| #28 "Japanese medicine" [TIAB] |
| #29 "Korean medicine" [TIAB] |
| #30 "medicinal herb" [TIAB] |
| #31 "oriental medicine" [TIAB] |
| #32 "traditional medicine" [TIAB] |
| #33 “alternative medicine” [TIAB] |
| #34 “complementary medicine” [TIAB] |
| #35 “Kampo medicine” [TIAB] |
| #36 “traditional Chinese medicine” [TIAB] |
| #37 “traditional Korean medicine” [TIAB] |
| #38 “traditional Oriental medicine” [TIAB] |
| #39 botanic [TIAB] |
| #40 decoction [TIAB] |
| #41 herb [TIAB] |
| #42 "herbal preparation" [TIAB] |
| #43 "herbal extract" [TIAB] |
| #44 "Herbal Medicine" [TIAB] |
| #45 "Herbal therapy" [TIAB] |
| #46 Drugs, Chinese Herbal" [Mesh] |
| #47 "Plants, medicinal" [Mesh] |
| #48 "Medicine, Chinese Traditional" [Mesh] |
| #49 "Medicine, Kampo" [Mesh] |
| #50 "Medicine, Korean Traditional" [Mesh] |
| #51 "Medicine, traditional" [Mesh] |
| #52 Ethnobotany [Mesh] |
| #53 Phytotherapy [Mesh] |
| #54 Plant extracts [Mesh] |
| #55 "Complementary Therapies" [Mesh] |
| #56 Or/1-21 |
| #57 Or/22-55 |
| #58 56 and 57 |
| **The Cochrane Library** |
| #1 ("Ambient Particulate Matter"):ti,ab,kw (Word variations have been searched) |
| #2 ("Asian dust"):ti,ab,kw (Word variations have been searched) |
| #3 ("coarse particle"):ti,ab,kw (Word variations have been searched) |
| #4 (Dust):ti,ab,kw (Word variations have been searched) |
| #5 ("Fine dust"):ti,ab,kw (Word variations have been searched) |
| #6 ("Airborne Particulate Matter"):ti,ab,kw (Word variations have been searched) |
| #7 ("Fine particle"):ti,ab,kw (Word variations have been searched) |
| #8 ("particular matter"):ti,ab,kw (Word variations have been searched) |
| #9 ("Particulate Air Pollutants"):ti,ab,kw (Word variations have been searched) |
| #10 ("Particulate Matter"):ti,ab,kw (Word variations have been searched) |
| #11 ("Particulate Matter, Airborne"):ti,ab,kw (Word variations have been searched) |
| #12 ("Particulate Matter, Ambient"):ti,ab,kw (Word variations have been searched) |
| #13 (PM0.1):ti,ab,kw (Word variations have been searched) |
| #14 (PM10):ti,ab,kw (Word variations have been searched) |
| #15 (PM2.5):ti,ab,kw (Word variations have been searched) |
| #16 ("Pollutants, Particulate Air"):ti,ab,kw (Word variations have been searched) |
| #17 ("Suspended particle"):ti,ab,kw (Word variations have been searched) |
| #18 ("ultrafine particle"):ti,ab,kw (Word variations have been searched) |
| #19 (ultrafine particulate):ti,ab,kw (Word variations have been searched) |
| #20 (Yellow dust):ti,ab,kw (Word variations have been searched) |
| #21 (suspended particulate matter):ti,ab,kw (Word variations have been searched) |
| #22 {or #1-#21} |
| #23 ("chinese drug"):ti,ab,kw (Word variations have been searched) |
| #24 ("chinese formulation"):ti,ab,kw (Word variations have been searched) |
| #25 ("chinese herb"):ti,ab,kw (Word variations have been searched) |
| #26 ("chinese medicine"):ti,ab,kw (Word variations have been searched) |
| #27 ("chinese plant"):ti,ab,kw (Word variations have been searched) |
| #28 ("chinese prescription"):ti,ab,kw (Word variations have been searched) |
| #29 ("Japanese medicine"):ti,ab,kw (Word variations have been searched) |
| #30 ("Korean medicine"):ti,ab,kw (Word variations have been searched) |
| #31 ("medicinal herb"):ti,ab,kw (Word variations have been searched) |
| #32 ("oriental medicine"):ti,ab,kw (Word variations have been searched) |
| #33 ("traditional medicine"):ti,ab,kw (Word variations have been searched) |
| #34 (“alternative medicine”):ti,ab,kw (Word variations have been searched) |
| #35 (“complementary medicine”):ti,ab,kw (Word variations have been searched) |
| #36 (“Kampo medicine”):ti,ab,kw (Word variations have been searched) |
| #37 (“traditional Chinese medicine”):ti,ab,kw (Word variations have been searched) |
| #38 (“traditional Korean medicine”):ti,ab,kw (Word variations have been searched) |
| #39 (“traditional Oriental medicine”):ti,ab,kw (Word variations have been searched) |
| #40 (botanic):ti,ab,kw (Word variations have been searched) |
| #41 (decoction):ti,ab,kw (Word variations have been searched) |
| #42 (herb):ti,ab,kw (Word variations have been searched) |
| #43 ("herbal preparation"):ti,ab,kw (Word variations have been searched) |
| #44 ("herbal extract"):ti,ab,kw (Word variations have been searched) |
| #45 ("Herbal Medicine"):ti,ab,kw (Word variations have been searched) |
| #46 ("Herbal therapy"):ti,ab,kw (Word variations have been searched) |
| #47 ("Drugs, Chinese Herbal"):ti,ab,kw (Word variations have been searched) |
| #48 ("Plants, medicinal"):ti,ab,kw (Word variations have been searched) |
| #49 ("Medicine, Chinese Traditional"):ti,ab,kw (Word variations have been searched) |
| #50 ("Medicine, Kampo"):ti,ab,kw (Word variations have been searched) |
| #51 ("Medicine, Korean Traditional"):ti,ab,kw (Word variations have been searched) |
| #52 ("Medicine, traditional"):ti,ab,kw (Word variations have been searched) |
| #53 (Ethnobotany):ti,ab,kw (Word variations have been searched) |
| #54 (Phytotherapy):ti,ab,kw (Word variations have been searched) |
| #55 (Plant extracts):ti,ab,kw (Word variations have been searched) |
| #56 ("Complementary Therapies"):ti,ab,kw (Word variations have been searched) |
| #57 {or #23-#56} |
| #58 #22 AND #57 |
| **EMBASE** |
| #1 'ambient particulate matter' |
| #2 'asian dust'/exp OR 'asian dust' |
| #3 'coarse particle' |
| #4 'dust'/exp OR dust |
| #5 'fine dust' |
| #6 'airborne particulate matter'/exp OR 'airborne particulate matter' |
| #7 'fine particle'/exp OR 'fine particle' |
| #8 'particular matter' |
| #9 'particulate air pollutants' |
| #10 'particulate matter'/exp OR 'particulate matter' |
| #11 'particulate matter, airborne' |
| #12 'particulate matter, ambient' |
| #13 pm0.1 |
| #14 'pm10'/exp OR pm10 |
| #15 pm2.5 |
| #16 'pollutants, particulate air' |
| #17 'suspended particle' |
| #18 'ultrafine particle'/exp OR 'ultrafine particle' |
| #19 ('ultrafine'/exp OR ultrafine) AND particulate |
| #20 ('yellow'/exp OR yellow) AND ('dust'/exp OR dust) |
| #21 suspended AND particulate AND matter |
| #22 'suspended particulate matter'/exp OR 'suspended particulate matter' |
| #23 'yellow dust' |
| #24 'ultrafine particulate' |
| #25 'chinese drug'/exp OR 'chinese drug' |
| #26 'chinese formulation' |
| #27 'chinese herb'/exp OR 'chinese herb' |
| #28 'chinese medicine'/exp OR 'chinese medicine' |
| #29 'chinese plant' |
| #30 'chinese prescription' |
| #31 'japanese medicine'/exp OR 'japanese medicine' |
| #32 'korean medicine'/exp OR 'korean medicine' |
| #33 'medicinal herb'/exp OR 'medicinal herb' |
| #34 'oriental medicine'/exp OR 'oriental medicine' |
| #35 'traditional medicine'/exp OR 'traditional medicine' |
| #36 'alternative medicine'/exp OR 'alternative medicine' |
| #37 'complementary medicine'/exp OR 'complementary medicine' |
| #38 'kampo medicine'/exp OR 'kampo medicine' |
| #39 'traditional chinese medicine'/exp OR 'traditional chinese medicine' |
| #40 'traditional korean medicine'/exp OR 'traditional korean medicine' |
| #41 'traditional oriental medicine' |
| #42 botanic |
| #43 'decoction'/exp OR decoction |
| #44 'herb'/exp OR herb |
| #45 'herbal preparation'/exp OR 'herbal preparation' |
| #46 'herbal extract' |
| #47 'herbal medicine'/exp OR 'herbal medicine' |
| #48 'herbal therapy' |
| #49 'drugs, chinese herbal'/exp OR 'drugs, chinese herbal' |
| #50 'plants, medicinal'/exp OR 'plants, medicinal' |
| #51 'medicine, chinese traditional'/exp OR 'medicine, chinese traditional' |
| #52 'medicine, kampo'/exp OR 'medicine, kampo' |
| #53 'medicine, korean traditional'/exp OR 'medicine, korean traditional' |
| #54 'medicine, traditional'/exp OR 'medicine, traditional' |
| #55 'ethnobotany'/exp OR ethnobotany |
| #56 'phytotherapy'/exp OR phytotherapy |
| #57 ('plant'/exp OR plant) AND extracts |
| #58 'complementary therapies'/exp OR 'complementary therapies' |
| #59 'plant extracts'/exp OR 'plant extracts' |
| #60 {or #1-#24} |
| #61 {or #25-#59} |
| #62 #60 AND #61 |
